# CELF1 is Up-Regulated in Glioma and Promotes Glioma Cell Proliferation by Suppression of CDKN1B: Erratum

**DOI:** 10.7150/ijbs.82390

**Published:** 2023-01-12

**Authors:** Liang Xia, Caixing Sun, Qinglin Li, Fang Feng, Enqi Qiao, Limin Jiang, Bin Wu, Minghua Ge

**Affiliations:** Zhejiang Cancer Hospital, Hangzhou 310022, Zhejiang Province, P.R.C

There was an error in Figure 7A in our paper due to misplace of Flow Cytometry data images during the final preparation of manuscript. We apologize that this was not detected by us before publication. The data analyses and conclusion remain unchanged.

## Figures and Tables

**Figure 7:**
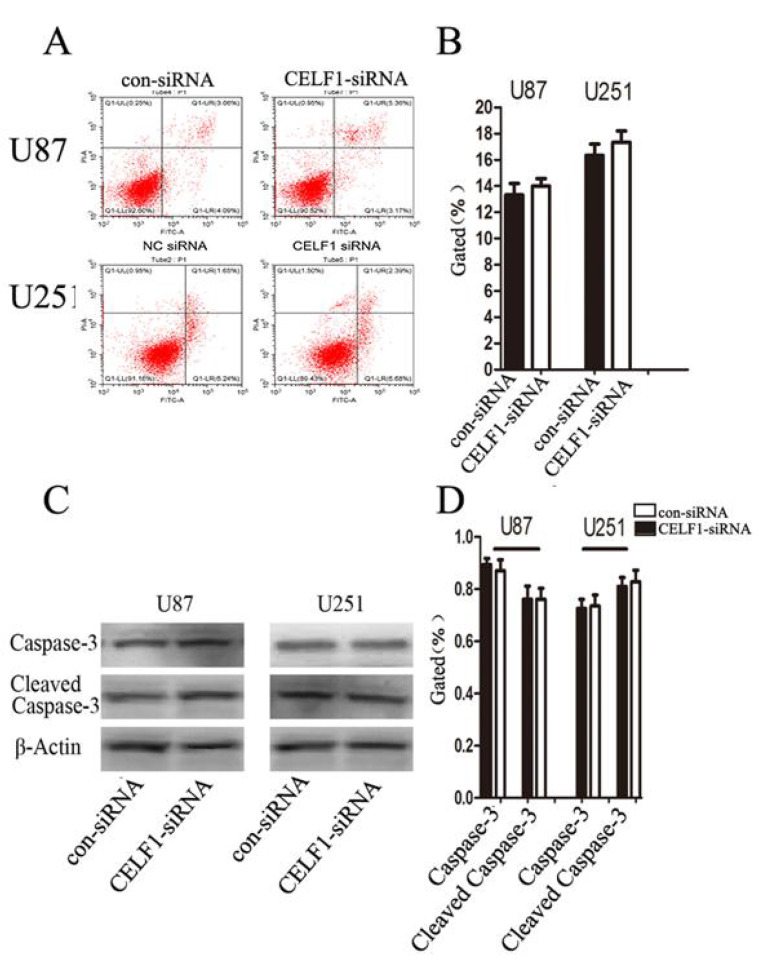
Correct Fig. 7.

